# Does Propolis Contain Tannins?

**DOI:** 10.1155/2014/613647

**Published:** 2014-05-14

**Authors:** Marco A. S. Mayworm, Carolina A. Lima, Augusto C. B. Tomba, Caroline C. Fernandes-Silva, Maria L. F. Salatino, Antonio Salatino

**Affiliations:** ^1^University of Santo Amaro, Rua Professor Enéas de Siqueira Neto 340, 04829-900 São Paulo, SP, Brazil; ^2^Department of Botany, Institute of Biosciences, University of São Paulo, Rua do Matão 277, 05508-090 São Paulo, SP, Brazil

## Abstract

Although polyphenols have been reported as common constituents of propolis, tannins have rarely been mentioned as its constituents. Propolis samples from seven localities in Brazil were analyzed for detection of proanthocyanidins (condensed tannins) and determination of the tannin content. Positive reaction for proanthocyanidins was observed for all samples tested. The contents of tannins varied in the range 0.6–4.1%. A high degree of correlation was noted between total phenols and tannin content. Red and green propolis contained high contents of tannins, while in brown propolis the content was lower. It is suggested that the contents of tannins should be a parameter to be considered in propolis characterization.

## 1. Introduction

Propolis is a resinous product collected by honeybees from buds or exudates of plants. The medicinal property of propolis was recognized in ancient times by several civilizations and in the last decades has gained great interest [1]. The wide diversity of activities has been reported for propolis and its constituents [2]. The composition of propolis is complex, comprising chiefly beeswax and resins of plant origin. Among the resin constituents, polyphenols such as flavonoids, phenylpropanoids, and benzophenones have often been reported [3, 4].

The composition of propolis resin varies with the geographic location and the plant source [5]. Brazilian green propolis is produced in the southeastern and central Brazil from vegetative buds of* Baccharis dracunculifolia* (alecrim-do-campo plant, Asteraceae). Prenylated phenylpropanoids [6] and caffeoylquinic acids [7] predominate in green propolis. Brazilian red propolis, from the littoral of northeastern Brazil, derives from trees of* Dalbergia ecastophyllum* (Leguminosae) and contains mainly isoflavonoids [8, 9].

Although phenolic compounds are common propolis constituents, scarce data [10] are available about tannins in propolis. Tannins are polyphenols that associate with proteins, forming stable complexes. The wide diversity of biological properties (antioxidant, antimicrobial, anti-inflammatory, antiallergy, and anthelmintic) has been ascribed to tannins [11, 12]. They are common in food and have been regarded as functional constituents [13]. Tannins precipitate food proteins, inhibit digestive enzymes, and may reduce the uptake of vitamins and minerals and thus may be digestive reducers and antinutritious. Two classes of tannins are recognized: hydrolyzable (gallic and/or ellagic), with restricted distribution in angiosperms, and proanthocyanidins (condensed tannins), which occur in most groups of angiosperms and other vascular plants [14].

The aim of the present investigation is to determine whether tannins (proanthocyanidins) occur in samples of different types of Brazilian propolis, to determine the contents of tannins, and to test if the contents of tannins and total phenolic substances are correlated.

## 2. Materials and Methods

**Figure 1:**
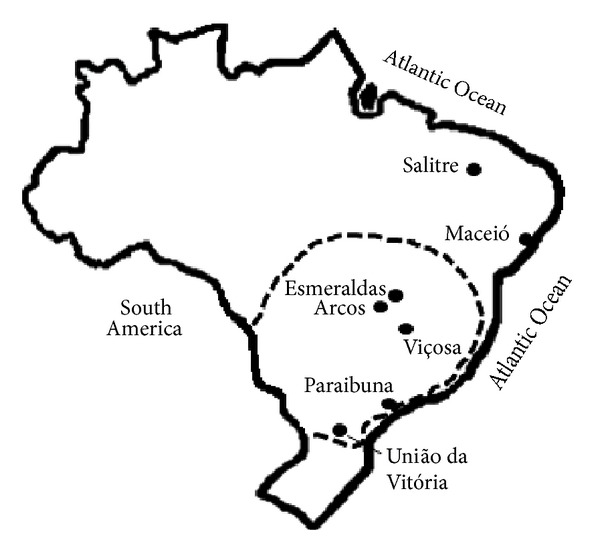
Localities of collection of samples of Brazilian propolis. Dotted line: approximate area of distribution of* Baccharis dracunculifolia* (alecrim-do-campo, Asteraceae), plant source of resin of green propolis.

The analyzed samples of propolis are listed in Table 1. They were collected in localities from the northeastern, southeastern, and southern regions of Brazil (Figure 1). The samples from the state of Minas Gerais (municipalities of Arcos, Esmeralda, and Viçosa; southeast) have strong characteristics of Brazilian green propolis: resinous odor, dark green color, and friable texture. The sample of Paraibuna (state of São Paulo; southeast) is also green, but darker than the samples from Minas Gerais. The sample from Maceió (state of Alagoas; northeast) is deep red, hard, and friable. The samples from União da Vitória (state of Paraná, South) and Salitre (state of Ceará; northeast) are brown, moldable, and sticky.

A portion of 1 g of each sample was ground in liquid N_2_ with mortar and pestle. The powdered samples were extracted four times with 50% ethanol on steam bath at 60°C during 30 min. The extracts were pooled and made up to the final volume of 50 mL. Extraction of all samples was done in triplicate.

Characterization of proanthocyanidins was done with 5 mL of the extracts by the acid/*n*-butanol hydrolysis method [15]. The contents of total phenolic substances were determined by the Folin-Ciocalteau method [16], using *p-*coumaric acid as reference compound. The content of tannins was determined using tannic acid as reference, by the method of precipitation with BSA (bovine serum albumin), dissociation of the complex, and colorimetric measurement at 510 nm, after addition of ferric chloride solution [17]. All analytical procedures were performed in triplicate. The Spearman nonparametric test [18] was conducted to evaluate the degree of correlation between the contents of total phenolic substances and tannins.

## 3. Results

All samples gave positive reaction for proanthocyanidins. Stronger reactions were obtained with the samples of green and red propolis. The highest content of tannins (4.1%) was obtained with the red propolis. The green propolis from Esmeralda and Viçosa come next, while the samples from Arcos and Paraibuna (also green) have nearly 2% tannins. Both samples of brown propolis (from Paraná and Ceará) have the lowest tannin contents (0.6–1.0%; Table 1). The green samples from the state of Minas Gerais have the highest contents of total phenolic substances (21% on average, Table 1). The samples from São Paulo have nearly half as much phenols, while the sample of red propolis has an intermediate content; the brown samples from the states of Paraná and Ceará have the lowest contents (3.9% and 6.5%, resp.; Table 1). The Spearman nonparametric test indicated high positive correlation between the contents of total phenols and tannins (*r* = 0.976).

## 4. Discussion

Considering the complex composition of propolis, the number of constituents reaching up to 300 [3], contents of tannins in the range 1–4% (Table 1) should be regarded as substantial. The results indicate that Brazilian red and green propolis contain higher tannin content, while the brown types lie in a lower rank. Wider samplings are needed, however, before definitive conclusions may be drawn.

The highest content of tannins found in the present work corresponds to red propolis (Table 1), produced with resin from a legume tree. Tannins are abundant in many legume tree species, such as* Stryphnodendron adstringens *(barbatimão, native to Brazil [19]) and* Acacia mearnsii* (wattle, Australia [20]). The plant source of green propolis (*Baccharis dracunculifolia*) belongs to Asteraceae, a family of herbaceous and shrubby species, usually with low or virtually null tannin content. However, the present results indicate that the resin of* B. dracunculifolia* contains substantial amounts of proanthocyanidins. Green propolis with high tannin contents (Esmeralda, Viçosa, and Arcos) came from the core region of* B. dracunculifolia* distribution (Figure 1), while the green propolis with low tannin content (from Paraibuna) came from a locality on the border of the distribution of* B. dracunculifolia*, as well as the brown propolis from União da Vitória (Figure 1). Seemingly, they contain either null or low quantities of* B. dracunculifolia* resin.

Despite the high positive correlation between total phenols and tannins, the proportion of tannins in the total phenolic content varies among the samples studied (Table 1). The high proportion of tannins in the sample of red propolis (Maceió) is coherent with the higher content of tannins in Leguminosae (source of red propolis), in comparison with Asteraceae (source of green propolis).

Tannins in food may have either positive or negative nutritious effects [21]. The relevant biological activities ascribed to tannins suggest that they should be a point of concern in propolis research. The contents of tannins may turn out a useful parameter in the characterization of propolis type. The samples from União da Vitória and Paraibuna contain marker substances of Brazilian green propolis (data not shown) and thus may be regarded as belonging to the green type, but both have low contents of tannins (Table 1). Several parameters have been suggested for standardization of propolis [3, 16]. The present results suggest that the content of tannins might be a parameter to be considered in this regard.

## 5. Conclusions

Tannins are common constituents of Brazilian propolis and the red and green types contain relatively high contents. A positive correlation exists between the contents of tannins and total phenols in propolis. The brown types from the southern and northeastern Brazil also contain tannins, but in lower contents. The content of tannins might come up as a parameter for propolis characterization.

## Figures and Tables

**Table 1:** Samples of Brazilian propolis, corresponding types, and contents (%) of total phenols and tannins (as determined by the method of BSA precipitation/colorimetry).

Municipality (state)	Propolis type	Total phenols	Tannins	Tannins/total phenols
União da Vitória (Paraná)	Brown	3.8	0.7	0.18
	Brown	4.0	0.6	0.15
Salitre (Ceará)	Brown	6.5	1.0	0.15
Paraibuna (São Paulo)	Green	10.6	2.1	0.20
Arcos (Minas Gerais)	Green	17.8	2.3	0.13
Esmeralda (Minas Gerais)	Green	25.5	3.6	0.14
Viçosa (Minas Gerais)	Green	19.5	3.6	0.18
Maceió (Alagoas)	Red	14.6	4.1	0.28
